# L’ostéoblastome de la cheville: une localisation exceptionnelle

**DOI:** 10.11604/pamj.2018.29.164.14198

**Published:** 2018-03-20

**Authors:** Redouane Hani, Mohamed Ben-aissi, Mohamed Saleh Berrada

**Affiliations:** 1 Service de Chirurgie Orthopédique, CHU Rabat, Hopital Ibn Sina, Université Mohammed V, Souissi, Maroc

**Keywords:** Tumeur bénigne, ostéoblastome, cheville, Benign tumor, osteoblastoma, ankle

## Abstract

L'ostéoblastome est une tumeur osseuse bénigne rare, qui survient chez l'adulte jeune avant 30 ans avec une nette prédominance masculine. Il se localise surtout au niveau du rachis et la diaphyse des os longs. La localisation au niveau de la cheville est exceptionnelle. Nous rapportons le cas d'un ostéoblastome de la cheville gauche chez un adulte révélé par des douleurs localisées et une impotence fonctionnelle partielle. La radiographie standard faite a été jugée strictement normale et devant la persistance des plaintes une TDM a été réalisée mais qui était trompeuse évoquant une ostéochondrite. C'est l'examen anatomopathologique effectué sur pièce opératoire qui a permis de redresser le diagnostic. Les suites chez ce patient ont été simples, le résultat fonctionnel était très satisfaisant. Notre observation est particulière par l'âge de survenue au-delà de 30 ans et par la localisation inhabituelle au niveau de la cheville. Même si des formes agressives ont été rapportées dans la littérature, le pronostic de l'ostéoblastome est bon, et les récidives peuvent être évitées par un traitement chirurgical adéquat.

## Abstract (en)

Osteoblastoma is a rare benign bone tumor occurring in adults younger than 30 years, with a clear predominance in men. It mainly occurs at the level of the spine and of the diaphysis in long bones. It occurs exceptionally in the ankle. We here report the case of an adult patient with osteoblastoma of the left ankle revealed by localized pain and partial functional disability. Standard radiographic evaluation was normal. Given the persistence of complaints CT scan was performed, but findings resulted in a misdiagnosis of osteochondritis. Anatomo-pathological examination of the surgical specimen allowed to adjust the diagnosis. Patient' s outcome was simple with very satisfactory functional result. Our case study is important due to the uncommon age of occurrence of the disease (patient older than 30 years) and to its unusual location at the level of the ankle. Even if aggressive forms have been reported in the literature, the prognosis of osteoblastoma is good and recurrences can be avoided by adequate surgical treatment.

## Introduction

L'ostéoblastome est une entité individualisée par Jaffe el Lichtenstein en tant que tumeur osseuse bénigne d'origine ostéoblastique caractérisée par la prolifération de nombreuses ostéoblastes et la présence d'une quantité importante de tissu ostéoïde au sein d'un stroma conjonctif richement vascularisé [1]. C'est une tumeur osseuse bénigne rare, représentant moins de 1% des tumeurs osseuse primitives et 3% des tumeurs bénignes, survenant souvent lors de la deuxième et de la troisième décennie avec une nette prédominance masculine [2]. Son siège préférentiel est le rachis 40% surtout au niveau de l'arc postérieur, suivi des os longs tel le fémur et tibia 20 à 30% et enfin les petits os des mains et des pieds avec une fréquence de 15% [2]. La localisation de cette tumeur au niveau de la cheville est exceptionnelle, ce qui fait l'intérêt de ce travail qui a été réalisé à partir d'un cas observé et suivi dans notre service.

## Patient et bservation

**Figure 1:**
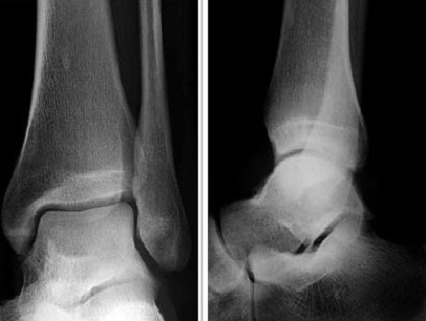
: Radiographies standards de la cheville gauche (préopératoire): normale

**Figure 2:**
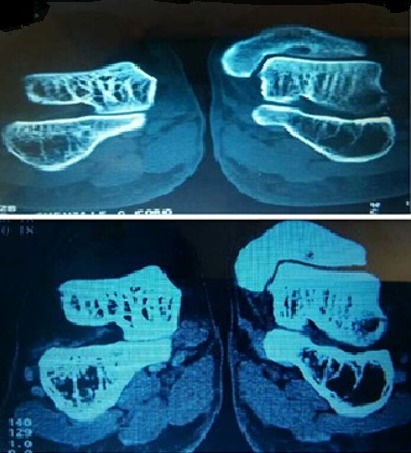
: Coupes TDM de la cheville gauche montrant la présence d’une image lacunaire antérieure sur la berge tibiale gauche

Nous rapportons le cas d´un patient de sexe masculin, âgé de 42 ans, sans antécédents particuliers, présentant depuis 3 mois une douleur localisée de la région antérieure de la cheville gauche intense et permanente, s'accentuant à la mobilisation et à la marche, résistante aux anti-inflammatoires non stéroïdiens et sans exacerbation nocturne, évoluant dans un contexte apyrétique avec conservation de l'état général. L'examen clinique retrouvait une douleur exquise à la palpation et à la mobilisation de la cheville avec une limitation des mouvements de flexion et extension par la douleur sans tuméfaction ni signes d'inflammation locaux. Une radiographie standard a été réalisée et a été jugée strictement normale (Figure 1). Devant la persistance des plaintes, une TDM a été faite et a montré une petite lacune au niveau de la partie antérieure du pilon tibial évoquant le diagnostic d'ostéochondrite (Figure 2). Une exérèse complète de la lésion a été réalisée et l'histologie a confirmé le diagnostic d'ostéoblastome. L'évolution pendant un an après était satisfaisante, avec disparition de la douleur, une reprise de l'appui après trois semaines et conservation d'une mobilité articulaire normale sans signe de récidive au bilan radiologique de contrôle.

## Discussion

L'ostéoblastome est une tumeur osseuse rare, représentant 1% des tumeurs primitives et 3% des tumeurs bénignes [3,4]. Il survient avant 30 ans dans 80% des cas, avec une nette prédominance masculine [2]. L'ostéoblastome se localise surtout au niveau du rachis dans 35 à 40% des cas [5,6], les os longs sont la deuxième localisation avec 20% (surtout au niveau de la diaphyse dans 80% des cas), la localisation au niveau de la main et du pied est exceptionnelle; seuls quelques cas ont été rapportés dans la littérature [2]. Cliniquement, la douleur est le maître symptôme d'intensité variable, exagérée à la mobilisation et à la marche, d'abord intermittente puis continue avec des paroxysmes nocturne et habituellement calmé par la prise d'acide salicylique ce qui oriente sensiblement le diagnostic [2]. La tuméfaction locale et la limitation du mouvement peuvent être observées mais elles sont moins fréquentes. La radiologie standard dans les formes typiques montre une petite zone lytique au sein d'une réaction corticale condensant d'intensité variable, ses contours ne sont pas toujours nets, parfois marqués par une ostéosclérose réactionnelle [7]. La TDM donne une analyse fine des lésions osseuses et des expansions tumorales extra osseuses et reste l'examen de base dans le diagnostic de l'ostéoblastome [2]. Du point de vue anatomopathologique, l'ostéoblastome se présente macroscopiquement comme un tissu compacte rougeâtre, hémorragique, friable et granuleux, microscopiquement il s'agit de tissu très vascularisé fait d'os immature et tissu ostéoïde, avec au niveau cellulaire de nombreux ostéoblastes, cellules géantes et quelques ostéoclastes, l'ensemble baigne dans abondant tissu conjonctif. La forme classique présente peu d'ostéogenèse réactionnelle périphérique et envahie peu les tissus mous. Il est important de noter que cet aspect histologique est comparable à celui de l'ostéome ostéoïde et que la frontière entre les deux est délicate à fixer. Le diagnostic de certitude est basé sur le curetage-exérèse de la tumeur avec étude anatomopathologique de la pièce opératoire objectivant la présence de la tumeur ostéoblastique [2]. L'évolution spontanée est marquée par l'extension locale de la tumeur. Aucune régression spontanée n'a été rapportée dans la littérature. Après traitement chirurgical, le pronostic est bon, cependant, des récidives ont été rapportées, surtout en cas de curetage-comblement. Des formes agressives ont été rapportées dans la littérature [8], avec parfois une transformation maligne selon Dorfmann [9] dans 3% des cas en dehors de toute radiothérapie, voire l'apparition d'ostéoblastome malin métastatique pour Mitchell et Ackerman [10].

## Conclusion

La localisation de l'ostéoblastome au niveau du pied est exceptionnelle, la confirmation diagnostique est histologique. Après un traitement chirurgical bien suivi, l'évolution est marquée par l'indolence et l'absence de récidive.

## Conflits d’intérêts

Les auteurs ne déclarent aucun conflit d'intérêts.
